# Acute Effects of Particulate Air Pollution on the Incidence of Coronary Heart Disease in Shanghai, China

**DOI:** 10.1371/journal.pone.0151119

**Published:** 2016-03-04

**Authors:** Xiaofang Ye, Li Peng, Haidong Kan, Weibing Wang, Fuhai Geng, Zhe Mu, Ji Zhou, Dandan Yang

**Affiliations:** 1 Department of Environment Health, School of Public Health, Fudan University, Shanghai, China; 2 Shanghai Key Laboratory of Meteorology and Health, Shanghai Meteorological Service, Shanghai, China; 3 School of Public Health and Key Laboratory of Public Health Safety (Ministry of Education), Fudan University, Shanghai, China; 4 Fudan Tyndall Centre, Shanghai, China; University of Tennessee Health Science Center, UNITED STATES

## Abstract

**Introduction:**

Evidence based on ecological studies in China suggests that short-term exposure to particulate matter (PM) is associated with cardiovascular mortality. However, there is less evidence of PM-related morbidity for coronary heart disease (CHD) in China. This study aims to investigate the relationship between acute PM exposure and CHD incidence in people aged above 40 in Shanghai.

**Methods:**

Daily CHD events during 2005–2012 were identified from outpatient and emergency department visits. Daily average concentrations for particulate matter with aerodynamic diameter less than 10 microns (PM_10_) were collected over the 8-year period. Particulate matter with aerodynamic diameter less than 2.5 microns (PM_2.5_) were measured from 2009 to 2012. Analyses were performed using quasi-poisson regression models adjusting for confounders, including long-term trend, seasonality, day of the week, public holiday and meteorological factors. The effects were also examined by gender and age group (41–65 years, and >65 years).

**Results:**

There were 619928 CHD outpatient and emergency department visits. The average concentrations of PM_10_ and PM_2.5_ were 81.7μg/m^3^ and 38.6μg/m^3^, respectively. Elevated exposure to PM_10_ and PM_2.5_ was related with increased risk of CHD outpatients and emergency department visits in a short time course. A 10 μg/m^3^ increase in the 2-day PM_10_ and PM_2.5_ was associated with increase of 0.23% (95% CI: 0.12%, 0.34%) and 0.74% (95% CI: 0.44%, 1.04%) in CHD morbidity, respectively. The associations appeared to be more evident in the male and the elderly.

**Conclusion:**

Short-term exposure to high levels of PM_10_ and PM_2.5_ was associated with increased risk of CHD outpatient and emergency department visits. Season, gender and age were effect modifiers of their association.

## Introduction

Coronary heart disease (CHD), also called ischemic heart disease, could lead to sudden onset of life-threatening acute events and is associated with significant morbidity [1,2]. It is the leading cause of death in the world according to the 2010 Global Burden of Disease Study [3]. In China, the CHD prevalence has been increasing during the decades. It has reported that age-standardized mortality rate of CHD in Chinese population increased from 62.52 per 100 000 people in 1990 to 77.89 per 100 000 people in 2010, which meant there were over 900 thousand death due to CHD in 2010 [4]. In recent years, evidence of the increased risk of CHD development and exacerbation after exposure to ambient particulate matter (PM) has been provided in epidemiological studies [5–8]. Particulate air pollution is of similar importance as other well accepted triggers of myocardial infarction, one subtype of CHD, such as physical exertion when the exposure prevalence in the population is considered [7]. As PM exposure is ubiquitous for population, it has considerable public health relevance, especially in China where the particulate air pollution is much severe [9]. Although many studies have reported associations between short-term PM exposure and total cardiovascular mortality in several cities in China [10–15], there is very scarce evidence on CHD morbidity [16]. Only one study in Shanghai has evaluated the acute effects of PM_10_ (particulate matter less than 10μm in aerodynamic diameter) on emergency department visits due to CHD based on one hospital data [17], which requires more large-scale studies in China as there might be different effects and/or effect magnitudes from the results in developed countries with much lower level of ambient PM.

Therefore, we performed a time-series study to investigate the acute effects of ambient PM on outpatients and emergency department visits for CHD in people aged over 40 years in Shanghai, China.

## Methods

### Health data

We analyzed daily CHD morbidity data in people aged above 40 years in 2005–2012 from hospital outpatient and emergency department visits in Shanghai. Data was obtained from Shanghai Health Insurance Bureau (SHIB). SHIB is the government agency in charge of the Shanghai Health Insurance System (SHIS), which provides compulsory universal health insurance to the most permanent residents in Shanghai (the coverage rate was 89.1% in 2010) [18]. As CHD diagnosis was not recorded in the SHIS for outpatient and emergency department visits, it was inferred from the medicine prescribed in the medical record, which was used for the conventional treatment of angina pectoris and myocardial infarction (MI), such as nitrates (nitroglycerin, isosorbide dinitrate), calcium channel blockers (verapamil, diltiazem) and beta-blockers (metoprolol). Nitroglycerin has been widely used for the treatment of angina and MI. Studies have reported a reduced risk of death associated with early treatment with nitroglycerin [19,20]. Verapamil and diltiazem, as vasodilators, can restore coronary artery flow and myocardial perfusion efficiently [21,22]. Beta-blocker therapy has been recommended in the course of MI for patients who are not at high risk for complications. It has revealed that metoprolol is the most frequently used beta-blocker in MI patients in China [23]. Daily CHD counts were stratified by gender and age groups (41–65 years, and >65 years).

The Institutional Review Board at the School of Public Health, Fudan University, approved the study protocol (NO. 2012-03-0324) with a waiver of informed consent. Health information was released to us at aggregate level without any potential of identifying individual patients. There was no contact with patients for this study.

### Exposure data

Daily data of air pollution included PM_10_, particulate matter less than 2.5μm in aerodynamic diameter (PM_2.5_), sulfur dioxide (SO_2_), nitrogen dioxide (NO_2_) and ozone (O_3_). The daily average concentrations of PM_10_, SO_2_ and NO_2_ in 2005–2012 were obtained from Shanghai Environmental Monitoring Center. As PM_2.5_ and O_3_ were not routinely monitored as the criteria pollutants in China before 2013, the daily PM_2.5_ data in 2009–2012 and O_3_ data in 2007–2012 were got alternatively from Pudong Meteorological Service and Shanghai Center of Urban Environmental Meteorology, respectively. The daily O_3_ was the daily maximum values of 8-h running mean. Weather data in 2005–2012 was obtained from Shanghai Meteorological Service, including daily mean temperature and relative humidity.

### Statistical analysis

Health data and exposure data were linked by date for time-series analysis. As the daily counts of CHD outpatient and emergency department visits were approximately Poisson distributed, we used a Poisson regression (quali-likelihood) in generalized linear models to estimate the association between CHD morbidity and PM exposure. The moving averaged concentrations of PM on the same day and the day before (lag 01) were used to present the main results. Data was also stratified by season (defined as warm season, May-October; and cold season, November-April). We controlled for the long-term time trend with a natural cubic regression spline with 7 degrees of freedom (df) per year [24], and 3 df per season per year for season-stratified analysis. We also controlled for day of the week and public holiday with indicator variables, and for daily temperature and relative humidity with a natural cubic spline with 4 df for each. We then added PM_10_ or PM_2.5_ with a natural cubic spline in a single pollutant model. As the CHD risk increased monotonously with ambient PM concentration, a linear effect of PM was then assumed in the model. The model is as below:

$$LogE\left(Y_{t}\right)=Intercept+ns\left(time,df\right)+ns\left(temperature,4\right)+ns\left(humidity,4\right)+dow+holiday+\alpha PM_{t-1,t}$$

where *E(Y*_*t*_*)* represents the expected count of CHD morbidity on day *t*, *ns* is a natural cubic spline, PM_*t-1*,*t*_ indicates 2-day averaged concentrations of PM on day *t-1* and *t*. Besides the main results on lag 01, PM on a single day lagged from the current day (lag 0) to the previous 6 days (lag 1 to lag 6) were also analyzed to examine the lag structure. To test the stability of PM effects on CHD morbidity, we added one of the gaseous pollutants (SO_2_, NO_2_ and O_3_) into the single pollutant model. We also performed a sensitivity analysis by varying the df for the time trend (from 5 to 13 df per year) to control for seasonality in the models.

Data management and regression analysis were performed with R version 3.1.0 (The R Project for Statistical Computing, http://www.r-project.org).

## Results

In this study, we examined 604944 CHD outpatient and emergency department visits in people aged above 40 years. Table 1 summarizes daily CHD morbidity, PM_10_, PM_2.5_, temperature and relative humidity during 2005–2012. On average, there were 207 CHD patients visiting outpatients and emergency departments each day. Daily counts of CHD morbidity in the cold season were larger than those in the warm season. There were more female patients in the outpatient and emergency departments. Patients older than 65 years accounted for almost three quarters of the total CHD morbidity. The overall mean concentrations of PM_10_ and PM_2.5_ were 81.7 μg/m^3^ and 38.6 μg/m^3^, respectively. The concentrations of PM_10_ and PM_2.5_ were both higher in the cold season than those in the warm season. The mean temperature was 25.0°C in the warm season and 9.7°C in the cold season.

**Table 1:** Summary (mean ± SD) of daily CHD outpatient and emergency department visits, PM concentrations and weather conditions in Shanghai in 2005–2012.

		All seasons	Cold season	Warm season
CHD outpatient and emergency department visits (n)				
	All	207.0 ± 51.1	237.8 ± 48.3	176.7 ± 32.0
	Male	92.4 ± 24.9	107.0 ± 24.1	78.0 ± 15.5
	Female	114.6 ± 28.5	130.8 ± 27.0	98.7 ± 19.6
	41–65years	56.6 ± 12.7	61.9 ± 12.7	51.5 ± 10.5
	>65years	150.4 ± 43.7	175.9 ± 42.3	125.2 ± 27.4
PM_10_ (μg/m^3^)		81.7 ± 54.4	93.8 ± 60.1	70.0 ± 45.4
PM_2.5_ (μg/m^3^)^a^		38.6 ± 26.7	48.7 ± 29.3	28.9 ± 19.5
Temperature (°C)		17.4 ± 9.1	9.7 ± 5.6	25.0 ± 4.3
Relative Humidity (%)		69.5 ± 12.3	67.9 ± 13.8	71.1 ± 10.4

^a^PM_2.5_ data was in 2009–2012.

**Fig 1:**
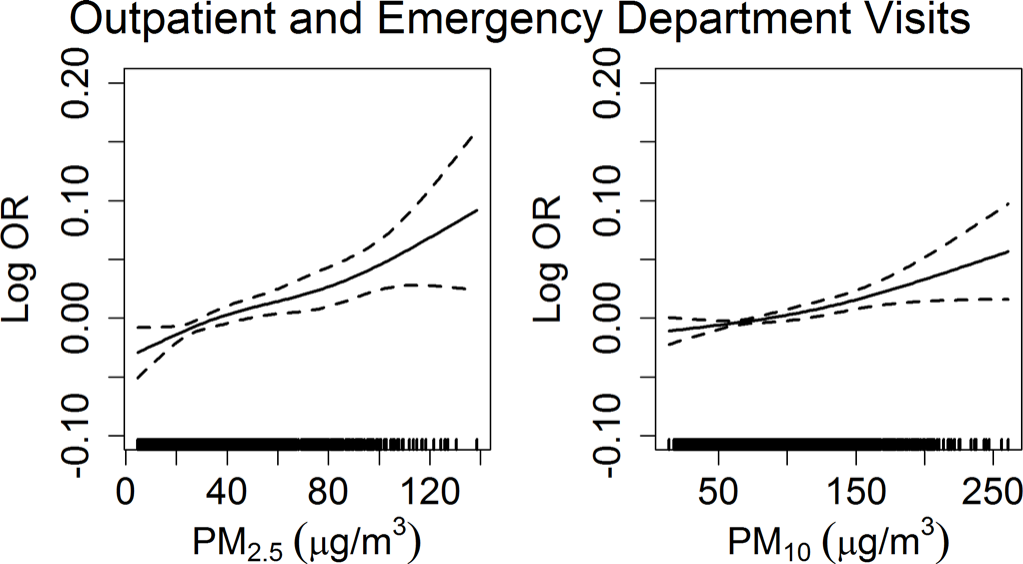
Smoothing plots of PM_2.5_ (left) and PM_10_ (right) against CHD outpatient and emergency department visits. X-axis is the PM concentrations at lag 01. The solid lines indicate the estimated mean percentage of change in daily outpatient and emergency department visits for CHD, and the dotted lines represent twice the standard error.

Fig 1 shows the relationship between PM at lag 01 and the total CHD morbidity. In general, CHD morbidity were positively related with both PM_2.5_ and PM_10_. An approximate linear effect of PM was found for CHD morbidity. CHD morbidity monotonously increased with the 2-day averaged concentrations of PM_2.5_ and PM_10_.

Table 2 presents the estimated percent of change in CHD morbidity for a 10 μg/m^3^ increase in 2-day averaged concentrations of PM_2.5_ and PM_10_ in different seasons. We found statistically significant associations between elevated PM exposure and increased number of CHD patients. A 10 μg/m^3^ increase in PM_2.5_ at lag 01 was associated with a 0.74% (95% CI: 0.44%, 1.04%) increase in CHD outpatient and emergency department visits, which was larger than that for PM_10_ with a 0.23% (95% CI: 0.12%, 0.34%) increase. There were different seasonal effects of PM on the total CHD morbidity. Significant PM effects were observed in the cold season, however, they were not statistically significant in the warm season. For instance, the estimate of the effect of PM_2.5_ on CHD outpatient and emergency department visits was largest in the cold season and equal to an estimated 0.93% (95% CI: 0.53%, 1.34%) increase in CHD risk per 10 μg/m^3^ increase in PM_2.5_ at lag 01 in the cold season, whereas it was nonsignificant in the warm season. Table 2 also indicates the PM effects on CHD morbidity in patients of different demographic factors. Gender and age could modify the association between PM exposure and CHD risk. The greater effect estimates were observed in men and people older than 65 years. In the cold season, there was obvious evidence of PM effects on increased risk of CHD morbidity except in people aged 41–65 years old for PM_10_. In the warm season, the effects of both PM_2.5_ and PM_10_ were only found in the male and the elderly.

**Table 2:** Estimated percent difference in CHD outpatient and emergency department visits (95% CI) in association with a 10 μg/m^3^ increase in PM_2.5_ and PM_10_ at lag 01 by season, gender and age group.

Group	All seasons		Warm season		Cold season	
	PM_2.5_	PM_10_	PM_2.5_	PM_10_	PM_2.5_	PM_10_
All	0.74 (0.44, 1.04)*	0.23 (0.12, 0.34)*	0.40 (-0.15, 0.95)	0.17 (-0.01, 0.35)	0.93 (0.53, 1.34)*	0.28 (0.12, 0.43)*
Male	1.10 (0.72, 1.47)*	0.33 (0.19, 0.47)*	1.05 (0.35, 1.75)*	0.28 (0.05, 0.52)*	1.25 (0.78, 1.73)*	0.38 (0.20, 0.57)*
Female	0.44 (0.10, 0.79)*	0.14 (0.01, 0.27)*	-0.13 (-0.76, 0.51)	0.08 (-0.14, 0.29)	0.67 (0.22, 1.13)*	0.19 (0.01, 0.36)*
41–65years	0.54 (0.09, 1.00)*	0.09 (-0.07, 0.26)	-0.28 (-1.04, 0.49)	0.01 (-0.26, 0.27)	0.79 (0.22, 1.37)*	0.13 (-0.09, 0.34)
>65years	0.80 (0.47, 1.13)*	0.27 (0.15, 0.40)*	0.64 (0.00, 1.29)*	0.24 (0.03, 0.45)*	0.97 (0.54, 1.41)*	0.33 (0.17, 0.50)*

* p<0.05.

**Fig 2:**
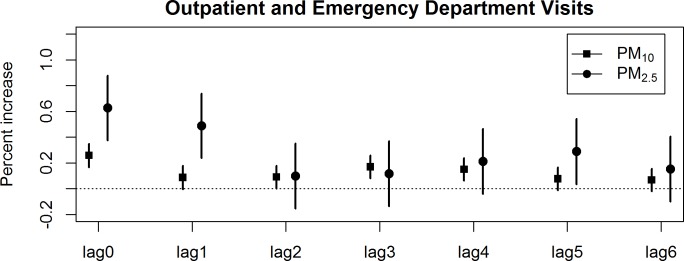
Estimated percent difference in CHD outpatient and emergency department visits (95% CI) in association with a 10 μg/m^3^ increase in PM_10_ and PM_2.5_ by lag days.

Fig 2 shows the estimated percentage of change in CHD risk for a 10 μg/m^3^ increase in PM_10_ and PM_2.5_ at different lag days. Across all seasons, we found that the estimates of the effects of PM_2.5_ and PM_10_ on CHD morbidity were largest at lag 0. There were increases of 0.63% (95% CI: 0.38%, 0.88%) and 0.26% (95% CI: 0.18%, 0.33%) in CHD risk for each 10 μg/m^3^ increase in PM_2.5_ and PM_10_ on the same day, respectively. CHD morbidity was also associated with PM_2.5_ at lag 1 and lag 5, with PM_10_ from lag 2 to lag 4.

Table 3 compares the results of single PM models with models adjusted for other gaseous pollutants. After controlling for SO_2_, NO_2_ or O_3_, the PM_2.5_ effects on CHD morbidity generally remained statistically significant, although the magnitude increased for SO_2_ and decreased for NO_2_. The estimates of effects of PM_2.5_ and PM_10_ changed slightly after O_3_ was added in the models. The PM_10_ effects on CHD morbidity tended to be insignificant after adjustment for SO_2_ and NO_2_. The choice of df for the time trend from 5 to 13 per year did not substantially affect the estimates of the effects of PM on CHD outpatient and emergency department visits (data not shown).

**Table 3:** Estimated percent difference in CHD outpatient and emergency department visits (95% CI) in association with a 10 μg/m^3^ increase in PM_2.5_ and PM_10_ with or without adjustment for gaseous pollutants.

Adjustment	PM_2.5_	PM_10_
Without adjustment	0.74 (0.44, 1.04)*	0.23 (0.12, 0.34)*
Adjusted for SO_2_	1.09 (0.63, 1.55)*	0.07(-0.07, 0.21)
Adjusted for NO_2_	0.59 (0.16, 1.02)*	-0.04(-0.18, 0.10)
Adjusted for O_3_	0.73 (0.42, 1.03)*	0.23 (0.10, 0.35)*

* p<0.05.

## Discussion

Although there are many studies investigated the association between ambient PM exposure and CHD morbidity and mortality in the US, the UK and other developed countries where PM concentration is quite low [25,26], evidence is limited with regard to the effects at higher levels of particulate air pollution. This study supports significant effects of both PM_10_ and PM_2.5_ on the acute onset and exacerbation of CHD, and also provides evidence of increased CHD risk at high elevated PM. This study, to our knowledge, is one of the few studies in China to evaluate the acute effects of PM on CHD morbidity [2,17,27,28].

Our results are mostly consistent with previous studies which reported significantly detrimental effects of particulate air pollution [6,26]. Studies in US and European cities have found high risk of CHD events due to recent PM exposure [1]. For example, Zanobetti et al [29] found an increase of 2.25% (95%CI: 1.10%, 3.42%) in MI admissions for a 10 μg/m^3^ increase in 2-day averaged PM_2.5_ concentration in 26 US communities. In our study, the effect estimate was slightly smaller, with only 0.74% (95% CI: 0.44%, 1.04%) increase in CHD outpatient and emergency department visits per 10 μg/m^3^ increase in PM_2.5_. The PM_2.5_ concentration was shown under 20μg/m^3^ in most US communities, almost half of the level in Shanghai in our study. The different effect magnitudes may be due to pollution level and population susceptibility in different locations, which was suggested in both US and China that the exposure-response function between cardiovascular mortality and PM exposure was relatively steep at very low levels of exposure and flattened out at higher exposure levels [30,31].

Two types of hospital visits, outpatient and emergency department visits, were combined together to be analyzed in this study. Their ratio was about 10%. Outpatient visits are usually not regarded as a good outcome in epidemiological studies of acute effects of air pollution in the US and Europe, where they are scheduled by appointment. However, situation is different in China. Outpatient visits are unscheduled as emergency department visits, and are first-come first-served. Patients could receive timely medical service in either outpatient or emergency departments. Therefore, the combined records of outpatient and emergency department visits in our study may have the potential to reflect the true morbidity information and offer opportunity to evaluate the acute effects of particulate air pollution.

An immediate impact on CHD of increased level of both PM_2.5_ and PM_10_ was found in the concurrent day (lag 0) and the previous day (lag 1), which was consistent with studies conducted in other locations showing a short time course [32,33]. We also observed the largest increase in CHD morbidity at lag 0 for PM exposure, which suggested an even shorter than daily timescale for PM effect on CHD incidence. Although it is impossible to assess the association on sub-daily level in this study, some studies have documented increased risk in hours after exposure to elevated PM [34,35].

Analyses by season indicated that effect estimates were higher in the cold season. A few studies found the modification of season or temperature on the PM-related effects on cardiovascular morbidity and mortality [36,37]. Our findings of a stronger association between particulate air pollution and cardiovascular risk in the cold season were consistent with previous studies in Shanghai [17,38]. The enhanced effects on ischemic heart disease in cool and dry weather were also indicated in Hong Kong [39]. One possible explanation is related to low temperature and increased blood pressure and viscosity in the cold season, which could be important causal factors in increasing winter morbidity and mortality due to heart attacks and strokes [40,41]. Another possible reason is the varying source and composition of PM in different seasons. In Shanghai, the prevailing wind direction is northwestern to northern in the cold season and southeast to east in the warm season, respectively [42]. The northern air flow transports high concentration PM_2.5_ emitted from coal burning for residential heating and biomass burning in the northern China in the cold season, while the eastern air flow which comes from the East China Sea carries clean air in summer [43]. For example, BC aerosol mass of non-local sources transported to Shanghai was highest in winter [44].

Stronger effects were observed in the elderly and male patients in the study. It is easy to understand the elderly are more vulnerable to particulate air pollution. Gender is usually treated as an effect modifier in air pollution epidemiology [45]. However results of previous studies on gender-specific effects of PM on CHD were conflicting [17,46]. Particulate air pollution showed higher CHD risk in the male in this study, which may be related to high exposure level of air pollution and tobacco smoking in men than women in China [47].

It is a challenge to elucidate the underling mechanisms of the PM effects on CHD, however, several possible mechanisms have been suggested in experimental studies and epidemiological studies. One potential mechanism is systemic inflammation, which is closely associated with cardiovascular diseases [48]. It has been shown that levels of inflammation markers such as high-sensitivity C-reactive protein increased significantly after exposure to particulate air pollution [49,50]. Increase in oxidative stress [51], endothelial dysfunction [52], blood viscosity [53,54] due to particulate exposure were also reported. They can accelerate the progression of atherosclerosis, cause the plaque instable, promote the formation of thrombosis, and increase the susceptibility to CHD. The other one potential mechanism is the impaired cardiac autonomic system as a result of particulate exposure. A reduced heart rate variability is usually treated as a marker of cardiac autonomic dysfunction. Studies on the association between short-term exposure to particulate air pollution and heart rate variability have supported an inverse relationship [55].

There are several limitations in this study. Firstly, the CHD records in outpatient and emergency department visits were not diagnosed according to ICD-10 but on medication prescription, which made misclassification with other cardiovascular diseases unavoidable, although the medicine was ascertained by several clinicians in hospitals. Hypertension could be included partly in this study as beta-blockers and calcium channel blockers have the effect of reducing blood pressure. Secondly, we used one monitoring data of PM_2.5_ for only 4 years, different from the city-wide PM_10_ data for 8 years. PM_2.5_ data from one monitoring station may has measurement error and introduce bias to our results.

In summary, in this time-series study coving large amount of CHD outpatient and emergency department visits in Shanghai during 2005–2012, we found that particulate air pollution (PM_10_ and PM_2.5_) was statistically significantly associated with increased CHD risk. Furthermore, the PM effects were higher in the cold season, the male and those over 65 years. These findings provide new evidence of high PM level on CHD incidence and may suggest further investigation on specific cardiovascular diseases in China.
